# Quantitative Comparison of Abundance Structures of Generalized Communities: From B-Cell Receptor Repertoires to Microbiomes

**DOI:** 10.1371/journal.pcbi.1005362

**Published:** 2017-01-23

**Authors:** Mohammadkarim Saeedghalati, Farnoush Farahpour, Bettina Budeus, Anja Lange, Astrid M. Westendorf, Marc Seifert, Ralf Küppers, Daniel Hoffmann

**Affiliations:** 1 Bioinformatics and Computational Biophysics, Faculty of Biology, University of Duisburg-Essen, Essen, Germany; 2 Center for Medical Biotechnology, University of Duisburg-Essen, Essen, Germany; 3 Institute of Medical Microbiology, Medical Faculty, University of Duisburg-Essen, Essen, Germany; 4 Institute of Cell Biology (Cancer Research), Medical Faculty, University of Duisburg-Essen, Essen, Germany; 5 Center for Computational Sciences and Simulation, University of Duisburg-Essen, Essen, Germany; 6 Center for Water and Environmental Research, University of Duisburg-Essen, Essen, Germany; University of Oxford, UNITED KINGDOM

## Abstract

The *community*, the assemblage of organisms co-existing in a given space and time, has the potential to become one of the unifying concepts of biology, especially with the advent of high-throughput sequencing experiments that reveal genetic diversity exhaustively. In this spirit we show that a tool from community ecology, the Rank Abundance Distribution (RAD), can be turned by the new MaxRank normalization method into a generic, expressive descriptor for quantitative comparison of communities in many areas of biology. To illustrate the versatility of the method, we analyze RADs from various *generalized communities*, i.e. assemblages of genetically diverse cells or organisms, including human B cells, gut microbiomes under antibiotic treatment and of different ages and countries of origin, and other human and environmental microbial communities. We show that normalized RADs enable novel quantitative approaches that help to understand structures and dynamics of complex generalized communities.

## Author Summary

Living things are parts of complex communities, similar to humans living in cities. A quantitative way of describing such communities is to measure the abundance of each species in the community so that a sorted list of abundance numbers is produced, a so-called Rank Abundance Distribution (RAD). With recent breakthroughs in genome analysis this approach can also be applied to very complex communities, such as the community of the myriads of microbes in a human gut (gut microbiome), or the diverse set of human immune cells. One problem with this approach is that it is not trivial to quantitatively compare RADs for different communities, especially if they are highly complex. We show that it is possible to computationally “normalize” RADs so that they can be quantitatively compared across many different communities. In this way, this normalization enables insight into structures and dynamics of arbitrary communities. We demonstrate this with applications to human immune cells, gut microbiomes under antibiotic treatment or under different nutritional regimes, and environmental microbiomes.

## Introduction

The *community*, i.e. the assemblage of organisms co-existing in a given space and time, is central to much of ecology [1], and since Darwin’s “entangled bank” [2] one of the great challenges of biology is to explain the observed species diversity in communities mechanistically as a consequence of interactions and evolution. Modern experimental methods of high-throughput sequencing have brought us closer to complete inventories of community diversity. Moreover, these methods enable us to widen the scope of the community concept to *generalized communities*, that we define as assemblages of genomically diverse entities, which include, apart from communities in classical ecology, for instance B or T cell repertoires of the adaptive immune system, viral quasi-species, tumors, or human microbiomes.

An intuitive description of a community composition is a table with columns *species* and *abundance*, possibly ordered from most to least abundant species (we use the term *species* here in a loose sense for operational taxonomic units or other genomically distinct biological entities). A visually more accessible graphical representation of this table would be a plot that arranges the species along the horizontal axis and the abundances as vertical bars, sorted from highest to lowest bar. While such a plot is expressive for a specific community, it does not lend itself to quantitative comparisons between communities. To illustrate this point, consider a comparison of a community of South-American animal species with one of Sub-Saharan African animal species from regions with otherwise similar conditions. The two species columns of our table would have practically no overlap so that a direct comparison of these tables or plots is not possible. The same lack of overlap has to be expected for other generalized communities. For instance if we compare high-throughput sequencing data of B cell receptors of two persons, it is unlikely that there are receptors on mature B cells that occur in both persons. Nevertheless, it is a meaningful biological question whether the abundance structures of the two sets of B cells differ, e.g. whether the B cell repertoire is dominated by a few clones with high cell numbers, or whether it is distributed over many different clones with low cell numbers.

A popular method for community comparison even in the absence of species overlap is to compute for each community a diversity index [3], i.e. a single number that characterizes one aspect of the community, for instance the species richness, the evenness of the distribution, the Shannon entropy, or one of many related measures [4], and then to compare the values of these indices between communities. The main disadvantage of this index approach is that it reduces a feature-rich abundance distribution to a single number, which may neglect important characteristics of that distribution.

**Fig 1:**
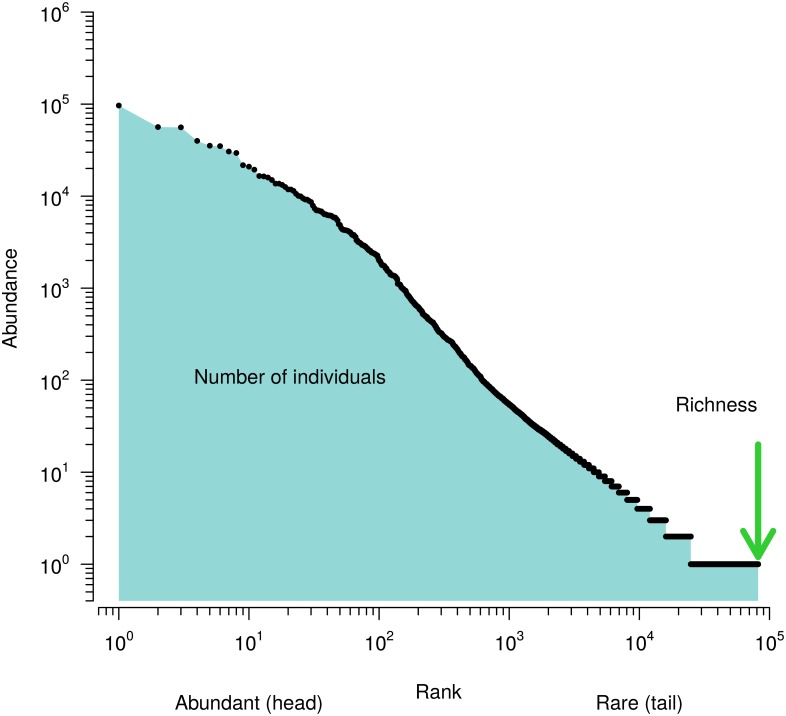
A typical Rank Abundance Distribution (RAD). A RAD with species abundances plotted in decreasing order from the most abundant (rank 10^0^ = 1) on the left to the least abundant species sampled from the community on the right. Both axes are scaled logarithmically to reveal the global structure of the RAD. Quantities such as the number of sampled individuals or the richness of the sample can be easily retrieved from the RAD.

An alternative approach that had a major influence on the development of the theoretical foundations of modern ecology is to discard the species labels of the species-abundance table, which then becomes a so-called Species Abundance Distribution (SAD; for excellent reviews see [5], or [6], chapter 9). There are several established ways of presenting the information contained in a SAD (see Fig 1 of [5]), for instance as straightforward histogram with species abundance as function of a species index, or as a binned histogram, typically with doubling bin widths with decreasing abundance. Here we focus on the Rank Abundance Distribution or RAD (Fig 1) as SAD representation. RADs are simply vectors of species abundances sorted in decreasing order, usually visualized as two dimensional plots, possibly with one or both axes scaled logarithmically. In comparison to the mentioned simple and binned histograms, RADs are more smooth due to their component sorting [7], and they retain the full biological resolution of the sampling experiment. The information content in RADs and probability distribution functions is the same, and one can be transformed into the other (see e.g. [8, 9]).

Obviously, RADs retain the complete shape information of species-abundance tables. The abstraction of species information means that RADs enable analysis of generic abundance distribution features of generalized communities, independently of the actual species composition.

RADs, and more generally SADs have been a key conceptual tool in the development and benchmarking of mechanistic models of ecological communities [10–15]. The mathematical functions resulting from mechanistic or statistical models, such as the log-normal distribution, were usually fitted to empirical RADs or SADs to identify the best community model. This basic research has paved the way for the application of these distributions to community comparisons, for instance to the characterization of community changes with changing environmental conditions (see e.g. [16, 17]. In these cases, the community comparison is typically a parameter comparison between fits of generic mathematical models to different RADs or SADs, or it is a purely visual comparison of these distributions [5].

In real-world samples, RADs are often not adequately described by a uniform mathematical model, e.g. a single log-normal distribution [18]. In macroecology, knowledge about the properties and relations between the observed animals and plants can be used to deconstruct multimodal distributions, and to fit simple models to fractions of the samples [3]. This knowledge is generally not available for high-throughput sequencing data of complex generalized communities, so that RAD analyses based on simple parametric models are difficult. This calls for a non-parametric approach. A further problem is that in practice the number of sampled species or sequences usually differs between samples. This means that RADs often have different dimensions and cannot be compared directly. It is possible to test for arbitrary pairs of RADs the null hypothesis that they originate from the same distribution using the Kolmogorov-Smirnov test [3, 19], but this is usually not helpful for quantitative comparisons. Technically, differences between a pair of RADs of different richness *m*, *n* could be also quantified by the Kolmogorov-Smirnov statistic *D* evaluated for the corresponding pair of cumulative distribution functions. However, it is problematic to interpret *D* in such cases since we are forced to equate non-reporting of |*m* − *n*| ranks in the shorter RAD with zero abundance, although the non-reporting may have technical reasons, e.g. limited sequencing depth. Thus, the question arises whether quantitative RAD comparisons are possible between samples of different richness. We quote from the widely cited SAD review by McGill *et al.* [5]:

“How do we compare SADs? Nearly all comparisons of SADs along gradients, deconstructions or time trajectories to date have been purely by visual inspection (…). Most particularly, these visual inspections have been performed on rank-abundance plots which, by using an x-axis that runs from 1 to S (i.e. species richness), seriously confounds the effects of species richness per se with other changes in the shape of the SAD (…). Changes in species richness are a legitimate factor that should be considered a change in shape of the SAD. However, changes in richness so strongly dominate in rank-abundance plots that no other changes are easily considered. Is there any other change in the shape of an SAD after controlling for the fact that productivity affects richness? We cannot say at the present time (…) More rigorous multivariate methods are needed.”

Here, we introduce MaxRank normalization of RADs, a new method that enables quantitative comparison of RADs, including their shapes. The approach is non-parametric and allows for the direct quantitative comparison of complex RADs without deconstruction and model fitting. An essential component of the method is the re-sampling of RADs up to a given richness. Consequently, the resulting normalized RADs (NRADs) are largely agnostic about the true richness of the original sample.

The fact MaxRank normalization uses re-sampling may lead to conflation with *rarefaction* and *rarefying* (on the distinction between the terms see [20]), two other techniques that also use re-sampling. *Rarefaction* [21] is typically used to estimate and compare richness between samples, i.e. exactly the quantity that is not of interest in MaxRank normalization. *Rarefying* (e.g. [22]) is applied to normalize OTU counts between samples. However, it is precisely the purpose of RADs and MaxRank normalization to abandon OTUs, and thus to make quantitative comparisons of abundance structures of different communities possible, irrespective of OTUs.

We show here that results from the quantitative comparison of normalized RADs reflect biological differences between samples. To emphasize the versatility of the method, we have chosen a diverse set of high-throughput sequencing data representing different types of generalized communities, namely, human B cell receptor repertoires, and various human and environmental microbiomes.

**Fig 2:**
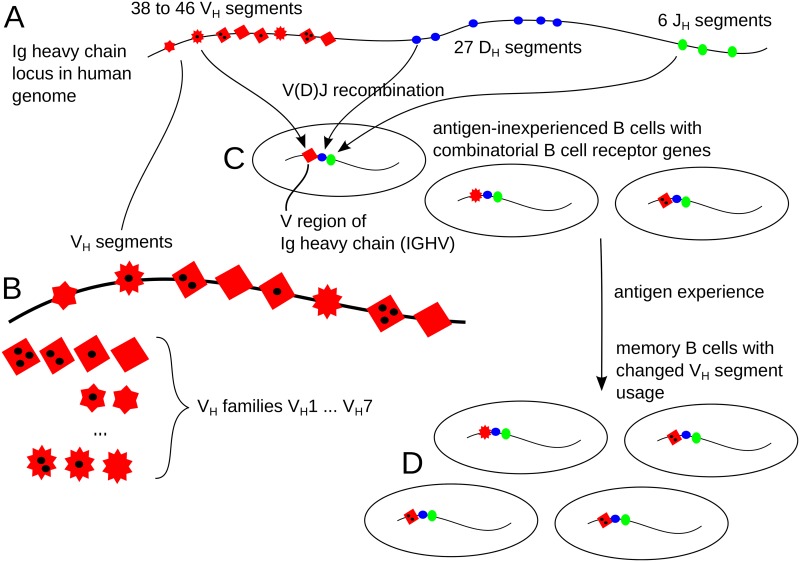
Diversity of the *V*_*H*_ region of BCRs. (A) The human genome contains sets of *V*_*H*_, *D*_*H*_, and *J*_*H*_ gene segments. (B) The “variable” *V*_*H*_ segments can be grouped into seven *V*_*H*_ families based on sequence similarity. (C) A genetically diverse pool of B cells is generated by V(D)J recombination. (D) Exposure to antigens induces an adaptation of the BCR repertoire, generating genetic variants and changing the usage pattern of *V*_*H*_ gene segments.

As one example of generalized communities we use human B cell receptor (BCR) repertoires. The diversity of BCRs in an individual is crucial for the recognition of antigens and the adaptive immune response [23]. Here we focus on the so-called *heavy* part of the receptor encoded by combinations of gene segments of the Ig heavy chain (IGHV) locus (Fig 2A; [24]), and especially on the diversity of the “variable” *V*_*H*_ segments in that part (Fig 2B). Based on sequence homology, the *V*_*H*_ segments are grouped into seven families (*V*_*H*_1—*V*_*H*_7), with members of a family having more than 80% sequence homology [25]. The sizes of the families vary from 1 (*V*_*H*_6 family) to 18–21 (*V*_*H*_3 family). The primary repertoire of rearranged IGHV genes among naïve, antigen-inexperienced B cells (Fig 2C) typically encompasses all available *V*_*H*_ segments, although abundances can vary considerably between *V*_*H*_ gene segments. Exposure to antigens leads to a selective adaptation of the BCR repertoire that results in individual-specific sets of memory B cells (Fig 2D). In the course of this complex maturation process, the usage of *V*_*H*_ segments in BCR rearrangement repertoires may change. On top of this layer of complexity, several *classes* of BCRs with different biological functions, such as IgG or IgM, are generated by class switching. Depending on the chronological order of these different processes, and on the individual immune histories, we can expect more similar or more divergent IGHV gene diversity between receptor classes and individuals in memory B cells. We study this question with RADs computed from High-Throughput Sequencing (HTSeq) data.

HTSeq technology is also transforming the study of microbial communities, because it allows us for the first time to see these complex assemblages in their full diversity [26, 27]. For instance, we now start to see the diverse composition of human gut microbiomes, and we begin to understand the links between the human microbiome, health and disease [28]. However, the deluge of data makes us also aware of the need for new ways to analyze and model such complex systems, e.g. with methods developed in ecology [29], such as RADs. We have selected three HTSeq data sets to demonstrate the potential and limitations of RADs for the analysis and the modeling of microbiomes: the considerable effect of antibiotics on gut microbiomes [30], a large collection of gut microbiomes from countries where different life styles prevail [31], and a diverse set of human and environmental microbiomes [32]. In these examples, we use RADs as an analytic tool to generate easily interpretable results, and as a basis for quantitative models.

## Materials and Methods

### Datasets

In the work presented here we used high-throughput sequencing (HTSeq) amplicon data from four different sources to compute and analyze NRADs, as described in the following.

#### B cell dataset

HTSeq amplicon data of IGHV genes were obtained for four different memory B cell fractions, *IgG*^+^*CD*27^−^ (for short: *IgG*^+^), *IgG*^+^*CD*27^+^, *IgM*^+^*IgD*^+^*CD*27^+^, $IgM_{only}^{+}CD27^{+}$, from two unrelated healthy donors, as published in [33] and in NCBI Sequence Read Archive (SRA) entry SRP062460. Since each fraction was split evenly into two independently processed subsamples, the total number of analyzed samples was 16. Sequence processing was described in [33]. Briefly, reads of bad quality were removed, and remaining reads collapsed to single sequences to eliminate PCR bias. Sequences were then assigned to their respective *V*_*H*_ gene segments. For all 16 samples, NRADs were computed using *V*_*H*_ gene segments as “species” and the number of distinct sequences assigned to each *V*_*H*_ gene segment as abundance of the respective species.

#### Gut microbiome dataset

HTSeq data (bacterial 16S rRNA gene fragment amplicons) from 528 human gut microbiomes [31] were retrieved from MG-RAST project 401 (http://metagenomics.anl.gov/). The dataset comprised 114 samples from rural Malawi, 315 samples from US metropolitan areas, and 99 samples from the Amazon region of Venezuela. Human subjects were aged between 11 days to 83 years with a median of 14 years.

#### GlobalPatterns dataset

Caporaso *et al.* [32] evaluated diversity patterns of microbial communities across a panel of HTSeq samples (bacterial 16S rRNA gene fragment amplicons) from diverse sources, including samples from human feces, skin, and tongue, environmental samples from ocean, estuary sediment, freshwater, soil, and three mock communities. Data from 26 of these samples were available through the R-package phyloseq [34], version 1.12.2. We used the OTU (Operational Taxonomic Unit) tables provided by phyloseq to compute NRADs. For better readability, samples were renamed according to the type of sample origin and given a consecutive sample number, e.g. from old names *LMEpi24M*, *SLEpi20M* in [32] to new names *lake1*, *lake2*. For human origins *tongue*, *palm*, *feces*, the same sample numbers refer to the same individuals, e.g. *tongue1* and *feces1* come from the same person number 1. A correspondence table linking old and new names is provided in S1 Table.

#### Antibiotic treatment dataset

Dethlefsen *et al.* [30] studied the effect of the antibiotic Ciprofloxacin on gut microbiomes of three healthy human individuals using HTSeq data (bacterial 16S rRNA gene fragment amplicons) before (8 measurements), during (4 measurements), and after treatment (6 measurements). The corresponding 18 OTU tables were retrieved from the supplementary material of [30].

### Computation of normalized RADs (NRADs)

**Fig 3:**
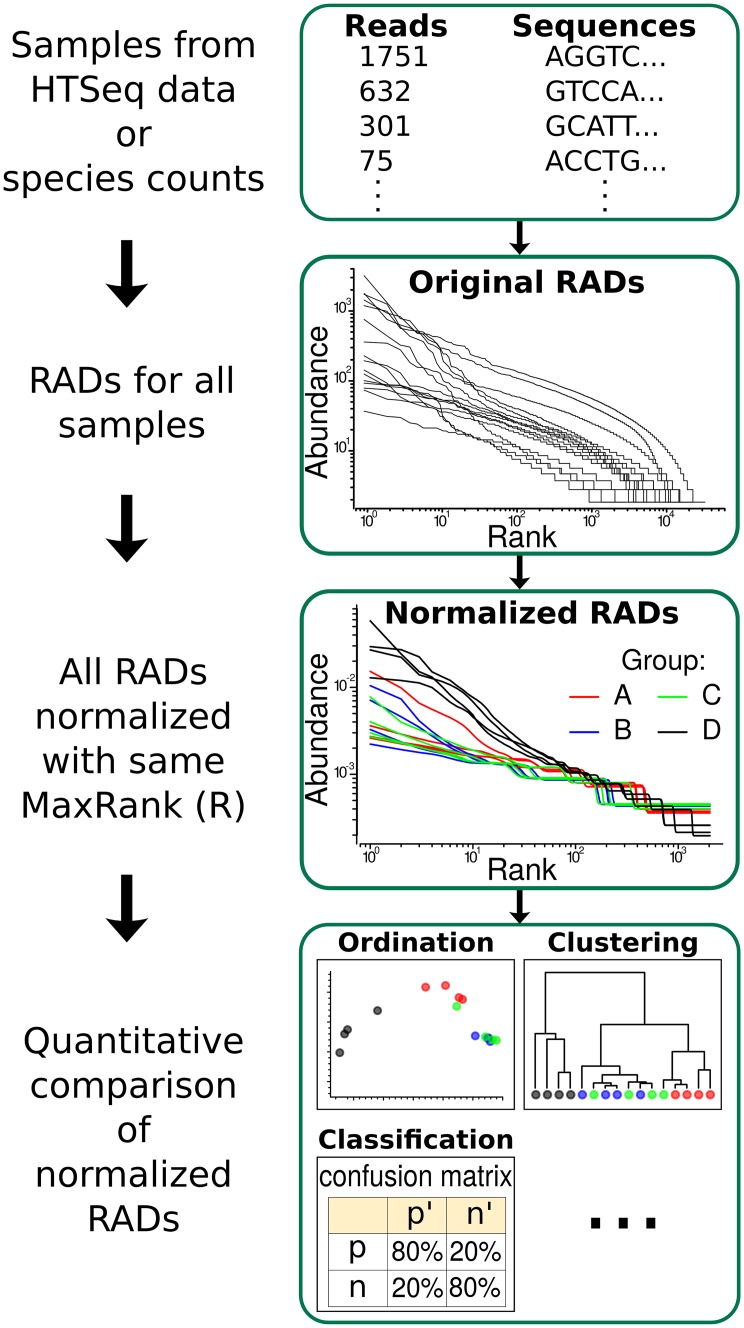
General process employed in this work. Flowchart of procedure from original species/abundances or sequence/reads data (top box) to original RADs, then to NRADs, and analyses based on NRADs.

For each dataset we followed the flowchart in Fig 3.

#### Compilation of abundance vectors

For each of the samples we had a two-column table of sequences and the corresponding numbers of sequence reads (here proxies for abundances), as obtained from the HTSeq experiment, or, equivalently, an OTU table with OTU names or numbers and the corresponding abundances in the sample. All data had been pre-processed and controlled for quality as described in [33], [30], [31], and [32], respectively.

The species columns were discarded, so that only a list or vectors of abundances remained for each sample. Abundances of each vector were sorted in descending order, bringing the highest abundance to the first element of the vector (= rank 1), the second highest to rank 2, etc. The resulting abundance vectors, one for each sample, were then normalized in the next step.

#### MaxRank normalization

MaxRank normalization is the key step to make abundance structures of generalized communities comparable, thus enabling all succeeding analyses. MaxRank normalization maps all rank abundance vectors to the same rank range from 1 to a common maximum rank *R*. The normalization procedure is explained in the following.

First we chose the maximum rank or “MaxRank” *R* (symbol *R* is used for the MaxRank throughout this work). The minimum *R* is 2 because *R* = 1 would eliminate all abundance structure. The maximum *R* is the minimum dimension of rank abundance vectors included in the analysis. For instance for the GlobalPatterns dataset, we had rank abundance vectors with dimensions from 2067 to 7679, and the maximum possible *R* for the whole set is therefore 2067. A value of *R* larger than this maximum would mean that we had to invent new ranks that have not been observed for at least one sample. In practice, this maximum *R* is often a good choice since it retains the abundance structure of all included communities in the greatest possible detail. We have therefore chosen in all analyses the maximum possible *R*.

Once the maximum rank had been fixed to a common *R* for all samples, we applied the MaxRank normalization separately to each sample. To this end we first generated for each sample *s* a pool of $N_{s}=\sum_{r=1}^{R_{s}}A_{sr}$ individuals, with *R*_*s*_ the original maximum rank (i.e. the richness) of sample *s*, and *A*_*sr*_ the abundance of rank *r* in that sample. From this pool we drew individuals at random with uniform probability and without replacement as long as the number of sampled ranks did not exceed *R*. In this way we generated a new, reduced abundance vector of *R* ranks, with a reduced number $N_{s}^{\prime }$ of individuals. Division of these reduced abundances by $N_{s}^{\prime }$ transforms the reduced abundance vector to a probability distribution (or relative abundances) for the *R* ranks with rank probabilities summing up to 1. We use therefore the terms *probability* and *abundance* in the remainder of the article as synonyms.

If *R* = *R*_*s*_, the procedure above reproduces exactly the original RAD. If *R* < *R*_*s*_, the random drawing of individuals from the pool in general introduces a sampling error in the abundances. To control this error, we repeated the procedure several times (typically 10–100 times) and averaged over all sampled abundance distributions. This average abundance distribution was returned as the final “normalized RAD” (NRAD) for each sample, together with 90% confidence intervals for the mean abundance at each rank, estimated as the interval between the 5% and 95% percentile of the bootstrapped averages at the respective rank.

For the NRAD of sample *i* we use as notation in the following a vector **a**_*i*_ of *R* abundances *a*_*ir*_:

$$a_{i}=\left(a_{i1},a_{i2},\ldots ,a_{iR}\right)$$ (1)

with elements *a*_*ir*_ ∈ ]0, 1] (ranks *r* = 1, 2, …, *R*) that are sorted (*a*_*ik*_ ≥ *a*_*i*ℓ_ for ranks *k* < ℓ), and normalized ($\sum_{r=1}^{R}a_{ir}=1$).

#### Software implementation of MaxRank normalization

We have implemented the methods used in this work in free open source software packages *RADanalysis* and *RankAbundanceDistributions* available in R (https://cran.r-project.org/web/packages/RADanalysis/) and Julia (https://github.com/DanielHoffmann32/RankAbundanceDistributions.jl).

### Analyses of NRADs

The last box of the flowchart Fig 3 indicates that sets of RADs normalized to a common *R* can be analyzed in numerous ways. In this article we used methods from three branches of data analysis: ordination, clustering, and classification. Since many ordination and clustering methods require a *distance* between the studied objects, we first describe how we computed distances between pairs of NRADs, and then the actual analysis methods.

#### Distances between NRADs

In this work, if not mentioned explicitly otherwise, a distance *d*_*R*_ between a pair of NRADs **a**_*i*_,**a**_*j*_ is the Manhattan distance:

$$d_{R}\left(a_{i},a_{j}\right)=\sum_{r=1}^{R}\left|a_{ir}-a_{jr}\right|.$$ (2)

The reason for using the Manhattan distance was that it accounts for NRAD-NRAD differences in a balanced way: NRADs typically show the largest differences in the first few ranks (in the “heads” of the NRADs, Fig 1), while the differences are typically small in the “tails” of the NRADs, which comprise many more ranks than the heads. The Manhattan distance gives the few large differences in the small heads and the many small differences in the large tails approximately the same weights.

All pairwise distances *d*_*R*_(**a**_*i*_, **a**_*j*_) between NRADs in a dataset were collected in a distance matrix for that dataset. The distance matrix was then used for distance based ordination and clustering.

We tested the practical suitability of the Kolmogorov-Smirnov statistic *D* as a distance measure using the function *ks.test* of R-package stats [35], version 3.2.2.

#### Ordination

We used classical multi-dimensional scaling (cMDS) [36] as an ordination method to arrange NRADs (Eq 1) of each dataset in an expressive and visually accessible way, usually in two dimensions called *first coordinate* and *second coordinate*. For a cMDS analysis, the distance matrix of the dataset to be analyzed was submitted to the *cmdscale* function of the R-package stats [35], version 3.2.2, or the *classical_MDS* function of Julia package MultivariateStats, release 0.1.0 (https://github.com/JuliaStats/MultivariateStats.jl).

#### Clustering

NRADs of the B cell and GlobalPattern data were clustered hierarchically by applying function *hclust* of R-package stats, version 3.2.2, to the NRAD distance matrix using the complete linkage cluster criterion.

#### NRAD averaging

Groups of NRADs, for instance the three groups of NRADs of gut microbiomes of individuals (a) before, (b) during, or (c) after treatment with Ciprofloxacin [30], or NRADs of gut microbiomes of individuals in certain age intervals [31] were summarized by computing an average NRAD ${\bar{a}}^{\left(g\right)}=\frac{1}{n}\sum_{i=1}^{n}a_{i}$ for each group *g* with members *i* = 1, 2, …, *n*. Note that averages of NRADs are NRADs themselves with the same MaxRank *R* and total abundance of 1. The 90% confidence interval for the mean NRAD of each group *g* was estimated for each rank, estimated as the interval between the 5% and 95% percentile of the bootstrapped averages at the respective rank.

#### Classification

For the classification of gut microbiomes according to country of origin we trained random forests [37] models with R-package randomForest, version 4.6-12 [38] with NRADs as predictors and country (MV vs. US) as class labels. The importance of each rank for the classification was estimated by computing the effect on the classification performance of randomly permuting class labels for each rank. Models were tested by threefold cross-validation, i.e. threefold training of a model on a randomly selected 2/3 of the data, followed by predicting the labels of the left-out 1/3, and comparison of predictions with ground truth from [31]. Prediction performance was quantified by the accuracy *ACC* and the *κ* statistic [39]:

$$ACC=\frac{n_{correct}}{N},$$ (3)

$$\kappa =\frac{ACC-ACC_{expect}}{1-ACC_{expect}},$$ (4)

with *N* predictions of which *n*_*correct*_ were correct, and *ACC*_*expect*_ the accuracy expected by randomly guessing from the given true distribution of labels with guessing probabilities as obtained from the model. For example, for *N* instances labeled by country *X* or *Y* we predict with the model *n*_*pred*,*X*_ and *n*_*pred*,*Y*_, while the true numbers are *n*_*true*,*X*_, *n*_*true*,*Y*_, and we have then *ACC*_*expect*_ = (*n*_*pred*,*X*_/*N*) ⋅ (*n*_*true*,*X*_/*N*) + (*n*_*pred*,*Y*_/*N*) ⋅ (*n*_*true*,*Y*_/*N*). We report *κ* because *ACC* can be biased if the different labels are not represented by approximately equal numbers of instances. A simplified interpretation of *κ* is the fraction of prediction accuracy that is not explained by guessing. A perfect model has *κ* = 1, a randomly guessing model *κ* = 0.

#### Model fitting

For the B cell data, geometric distributions were fitted to *V*_*H*_ gene sequence counts with a maximum likelihood method in Julia v0.4 package *Distributions* (https://github.com/JuliaStats/Distributions.jl) and tested for consistency by a one-sample Kolmogorov-Smirnov test in Julia v0.4 package *HypothesisTests* (https://github.com/JuliaStats/HypothesisTests.jl).

For the model of gut microbiome entropy as function of age, we computed Shannon entropies *H*_*R*_ from NRADs with function *entropy* from Julia v0.4 package *StatsBase* (https://github.com/JuliaStats/StatsBase.jl). Age was provided by the dataset given by [31].

The model was fitted to the set of (entropy, age) pairs by least-squares minimization with the Levenberg-Marquardt algorithm [40], as implemented in Julia v0.4 package *LsqFit* (https://github.com/JuliaOpt/LsqFit.jl). Starting conditions for the fit were the same for the MV and US set, namely $H_{R}^{0}=3.5$, *λ*_*R*_ = 0.19, $H_{R}^{max}=6.0$, obtained from a rough data-based estimate. Confidence intervals were estimated from the Jacobians at the optimally fitted parameters, as implemented in *LsqFit* function *estimate_errors*.

#### Comparison to standard distributions

Five standard distributions commonly used to model RADs were fitted to the RADs of the GlobalPattern set as described in [41] and implemented in the *radfit* function of R-package vegan, version 2.2-1 [42]: broken stick (null model, no free parameter), preemption (geometric series), log-normal, Zipf, and Mandelbrot. Fits to the Mandelbrot distribution did not converge, so that only the other four distributions are shown.

## Results

### B cell dataset: Abundance structures of biologically distinct generalized communities

With their highly diverse repertoire of antigen-binding receptors, human memory B cells are an example of what we have earlier termed *generalized community*. This diversity is achieved by a process that is only partly understood and currently subject of intense research [43, 44]: it starts with the genetic recombination of triplets of specific *V*_*H*_, *D*_*H*_, and *J*_*H*_ gene segments from genetic pools of these segments, followed by various mutation and selection steps, and eventually leads to distinct classes and sub-classes of memory B cells.

**Fig 4:**
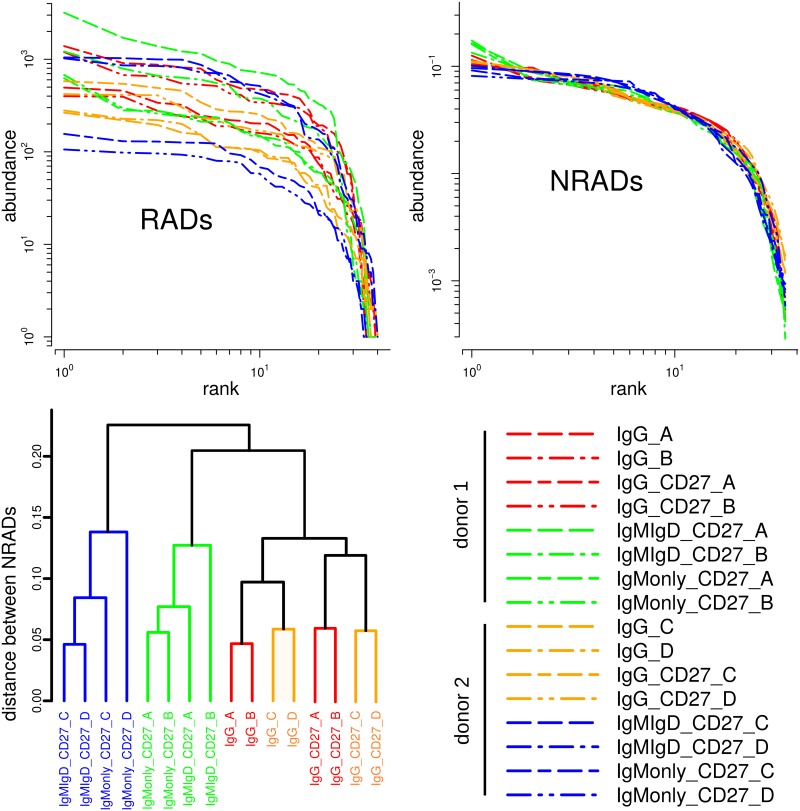
Rank abundance distributions of memory B cell receptors. Four different B cell receptor sub-classes from donors 1 (replicate samples A, B) and 2 (replicates C, D) are compared. Top left panel: Log-log plot of RADs prior to normalization. Top right: Log-log plot of corresponding NRADs. Legend for RADs and NRADs is given in bottom right panel. Bottom left: Hierarchical clustering tree based on all pairwise distances between the 16 NRADs.

We assume as a working hypothesis that all memory B cells underwent the same diversity generating process. If this is true, we should see a very similar *V*_*H*_ gene rearrangement pattern (Fig 2) in all sub-classes of memory B cells, leading to the same normalized RADs (NRADs) of memory B cells in all sub-classes. To test this hypothesis, we used HTSeq data of the immunoglobulin heavy-chain variable (IGHV) regions of four large memory B cell sub-classes, *IgG*^+^, *IgG*^+^*CD*27^+^, $IgM_{only}^{+}CD27^{+}$, *IgM*^+^*IgD*^+^*CD*27^+^, from two donors. The IGHV regions derive from the genomic pool of 38–46 *V*_*H*_ segments (Fig 2A) and have been modified by mutation and selection steps. If we interpret the original set of *V*_*H*_ segments as the “species” to be ranked according to their abundances, we should under our working hypothesis see the same NRADs in all memory B cell sub-classes. To exclude distortions of abundances due to primer bias, we collapsed abundances of *V*_*H*_ segments from the measured read numbers to the numbers of distinct *V*_*H*_ sequence variants. Thus, in this case an abundance is the number of distinct sequences that originate from the same *V*_*H*_ segment, and that have been diversified by somatic mutations and clonal expansions. Fig 4 summarizes the results.

The non-normalized RADs (top left of Fig 4) have similar, boomerang-like shapes, although direct comparisons is difficult since differences in abundance span more than one order of magnitude, and maximum ranks differ between 35 and 40. For direct comparison we therefore normalized the RADs to a MaxRank *R* = 35 (top right of Fig 4). The resulting NRADs have overall very similar shapes, lending support to our working hypothesis of a common generation and selection process. However, there are notable features that differentiate between groups of NRADs. For instance at rank 1 the most diverse IGHV regions in IgM receptors of donor 1 (green curves in Fig 4) are more abundant than all other rank 1 abundances. Conversely, for donor 2 the most diverse IGHV regions in IgM (blue curves) are the least abundant of all rank 1 abundances. Towards higher ranks, IgM abundances of both donors (blue and green) are more similar to each other. IgG receptors (red and orange) have more similar abundance structures throughout all ranks, and have stronger right tails than IgM receptors, indicating a more even *V*_*H*_ segment diversity in IgG than IgM receptors.

The differences between the NRAD curves are subtle, but they emerge clearly when we quantitatively analyze NRAD distances (Eq 2). In the hierarchical clustering tree of the distances (Fig 4) we see three main clusters of NRADs, a big cluster of *IgG*^+^ and *IgG*^+^*CD*27^+^ NRADs on the right of the tree in red and orange, and two clusters of IgM related NRADs. These three clusters appear robustly, no matter whether the average linkage or the complete linkage criterion is used for clustering.

As expected for replicates, (A, B) and (C, D) of the same sub-class coming from the same donor yield NRADs that are most similar and thus fall into the same lowest-level clusters. Beyond this, memory B cell sub-classes have remarkably different cluster structures. The big *IgG*^+^/*IgG*^+^*CD*27^+^ cluster (red and orange) of eight NRADs has a substructure of an *IgG*^+^ cluster and a separate *IgG*^+^*CD*27^+^ cluster, i.e. here the memory B cell sub-class has a stronger impact on the NRAD than inter-donor differences. This is different for the $IgM_{only}^{+}CD27^{+}$ and *IgM*^+^*IgD*^+^*CD*27^+^ clusters. There, each of the two donors forms a cluster of its own that combines both $IgM_{only}^{+}CD27^{+}$ and *IgM*^+^*IgD*^+^*CD*27^+^ NRADs, i.e. in these two IgM sub-classes, inter-personal differences in *V*_*H*_ diversity patterns are stronger than differences between sub-classes.

Our working hypothesis was that we have basically a single, diversity generating process for all memory B cell sub-classes, leading to the same NRADs for *V*_*H*_ gene rearrangement pattern in all sub-classes. Overall, our results are consistent with this big picture since all NRADs are variants of the same boomerang shaped template. However, the quantitative analysis of NRAD distances picked up differences that require refinements of this model. This is not surprising since some of the steps that potentially affect *V*_*H*_ rearrangement diversity, for instance class switching, cannot apply equally to all memory B cell sub-classes. What is surprising are the specific differences in *V*_*H*_ rearrangement diversity between IgM and IgG sub-classes, e.g. the structure of the IgG cluster discussed above suggests that there could be significantly different selection pressures towards the final memory B cells in the two sub-classes *IgG*^+^ (i.e. *IgG*^+^*CD*27^−^) and *IgG*^+^*CD*27^+^.

Recently, we have used the same HTSeq data for a detailed sequence-based analysis of the clonal composition and genealogy of memory B cells [33]. Although our current NRAD-based analysis disregards much of the information used in [33], results are consistent: First, the *V*_*H*_ gene rearrangement composition is mostly very similar across the studied sub-classes of memory B cells, in agreement with a shared generation process [45]. Second, *IgM*^+^*IgD*^+^*CD*27^+^ and $IgM_{only}^{+}CD27^{+}$ show almost the same *V*_*H*_ gene rearrangement diversity, likely due to their clonal relatedness as described in [33]. Third, there are significant and consistent differences between *IgG*^+^*CD*27^+^ and *IgG*^+^*CD*27^−^ with respect to mutation load in both donors, also in agreement with [46] or [47].

**Fig 5:**
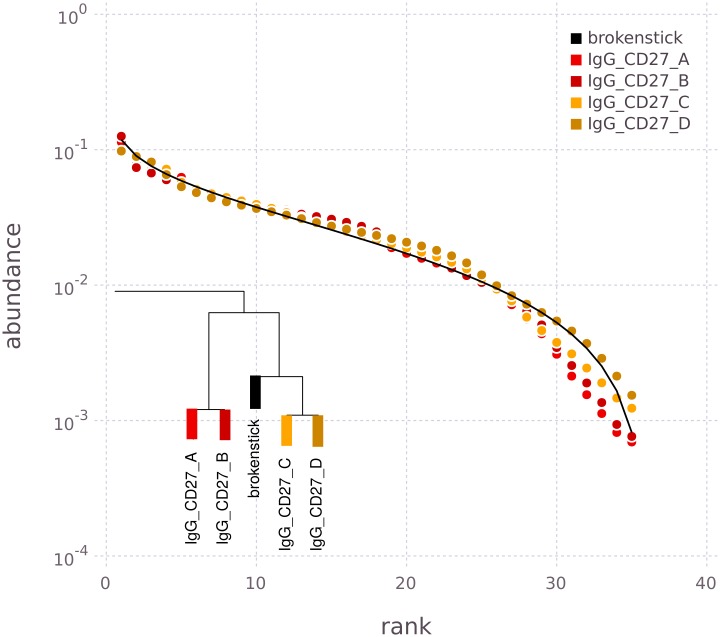
Broken stick distribution (solid line) and NRADs of *IgG*^+^*CD*27^+^ fractions (points). Inset: section of hierarchical clustering dendrogram where broken stick distribution appears. This plot adopts the usual presentation of the broken stick distribution in the literature with linear horizontal axis and logarithmic vertical axis. Therefore the boomerang shapes of the log-log Fig 4 appear horizontally stretched.

To conclude this section, we return to the conspicuous boomerang shape that is the template common to all B cell receptor RADs and NRADs (Fig 4). When testing for similarity to standard model distributions in ecology, we found that the broken stick distribution [13] is a good description for the NRADs of sub-class *IgG*^+^*CD*27^+^ (Fig 5). If included in the hierarchical clustering, the broken stick NRAD appears among the branches of the *IgG*^+^*CD*27^+^ sub-tree (inset of Fig 5). However, even for the other sub-classes, we cannot reject the broken stick distribution (p-values from Kolmogorov-Smirnov tests between 1.0 and 0.87 with a median of 0.99), though they deviate more than *IgG*^+^*CD*27^+^. Note that the normalized broken stick distribution in Fig 5 has no free parameters and therefore has not been fitted.

Several mechanisms in community ecology and elsewhere lead to broken stick RADs [13, 48–50]. A simple explanation for the observed RADs of *V*_*H*_ segment usage could be the following. Assume a fixed number $n_{V_{H}}$ of *V*_*H*_ segments in the genome, and a fixed total number *N*_*t*_ of all BCR sequence variants (i.e. summed over all *V*_*H*_ segments). The biological purpose of fixing *N*_*t*_ could be to provide a sufficient number of BCR variants to cover the typical antigen diversity. These two assumptions fix the average number $N_{t}/n_{V_{H}}$ of sequence variants per *V*_*H*_ segment. If this is all we know, the Maximum Entropy principle states that the geometric distribution is the most parsimonious explanation fulfilling these requirements [51]. The geometric distribution is the discrete equivalent of the continuous exponential distribution, which generates the broken stick RAD [48]. Thus, our argument posits a random process that produces numbers of sequence variants per *V*_*H*_ segment with a geometric distribution. In fact, sequence counts of most RADs are compatible with geometric distributions according to Kolmogorov-Smirnov tests at significance level 0.05 (exception: sample D of *IgM*^+^*IgD*^+^*CD*27^+^ with *p* = 0.04). Given that there is good agreement between the RADs of the BCR sub-classes (Fig 4), and also good agreement in the usage of individual *V*_*H*_ segments between human donors [33], our argument suggests the following two testable hypotheses. First, the random mechanism leading to the broken stick RAD could be encoded in the human genome and conserved among individuals with intact immune systems. Second, since our argument is generic, we should see the broken stick RAD also in other species with similar BCR rearrangement mechanisms.

### Antibiotic treatment dataset: Abundance structure reacts to perturbations

Dethlefsen *et al.* [30] reported the effects of a short course of Ciprofloxacin (Cp) treatment on the gut microbiomes of three healthy human individuals. When comparing gut microbiomes prior to treatment and during treatment, they found markedly perturbed taxonomic composition, richness, diversity, and evenness. These perturbations varied between individuals. After treatment, the community compositions recovered within four weeks to states close to pre-treatment, though with some species lost. We tested whether the dynamics of perturbation and recovery is reflected by changes of NRADs.

**Fig 6:**
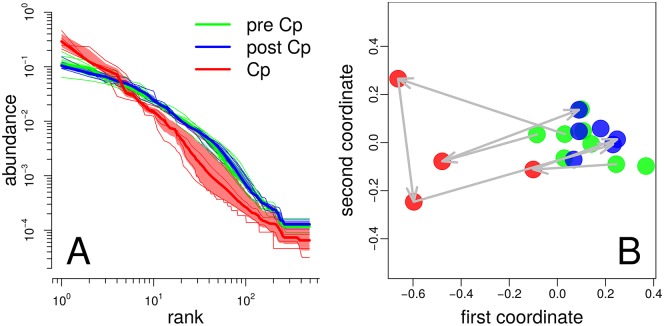
Abundance dynamics of gut microbiomes of three individuals under treatment with antibiotic Ciprofloxacin (Cp). (A) NRADs before (green), during (red), and after (blue) treatment. Bold lines are mean NRADs, shaded regions are 90% confidence intervals of the means. (B) MDS of NRADs with one point per NRAD using the same color code as in panel A. For each of the three individuals, arrows connect points corresponding to the last measurement before treatment, measurements during treatment, and the first measurement after treatment. The two coordinates of the MDS plot explain 89% of the NRAD distances.

The NRADs fall into two clusters, one well-defined “off-Cp” cluster of NRADs before and after treatment (blue and green in Fig 6), and one wider “on-Cp” cluster during treatment (red in Fig 6). All on-CP NRADs have heavier heads and less weight in the tails, consistent with the decreased gut microbiome diversity under treatment discovered by [30]. After normalization, we could compute distances between all pairs of NRADs and apply multidimensional scaling (MDS) to the distance matrix. The MDS plot Fig 6B captures the abundance dynamics from the well-defined off-Cp cluster on the right to the wider on-Cp cluster on the left and back again to the off-Cp cluster on the right. We usually find in MDS analyses a strong correlation of the first coordinate with Shannon entropy. Hence, the dynamics in the MDS plot Fig 6B from right (pre-Cp) to left (Cp) to right (post-Cp) corresponds to a succession of high-low-high entropy. This can also be seen directly from the NRADs: the off-Cp NRADs have a relatively heavy tail and weak head, i.e. a more even distribution with higher entropy, corresponding to a more diverse gut microbiome. Conversely, the on-Cp NRADs have a more heavy head and weaker tail, i.e. a less even distribution with lower entropy, corresponding to a less diverse gut microbiome, partly decimated by the effect of the antibiotic.

Dethlefsen *et al.* [30] remarked that after treatment several taxa failed to recover, while the participants in the study had normal intestinal function, and they argued that the eliminated taxa after treatment may have been replaced by other taxa with similar functions. The fact that pre- and post-Cp NRAD ensembles have the same shapes and form a single compact off-Cp cluster (Fig 6) supports this assessment.

It is instructive to compare our analysis based on the abundance structure with an OTU composition analysis as in Fig 6 of Ref [30]. The OTU based PCA in Ref [30] has a cluster structure that is influenced by both the individual microbiome donor and by the treatment state. The conflation of both influences makes the result of the PCA richer but also more difficult to interpret: If we consider individual microbiome compositions, all three individuals have different microbiome dynamics under treatment. Conversely, the NRAD based analysis is blind to individual differences in microbiome composition. This blindness to composition means on the other hand to focus on the abundance structure, which makes the result in our Fig 6 more easy to interpret: In terms of the abundance structure, all three individuals behave in the same way, clearly showing a generic effect of the antibiotic treatment.

### Gut microbiomes dataset: NRADs enable quantitative models

Yatsunenko *et al.* [31] found in 528 gut microbiomes from Malawi, United States and Venezuela, that (1) species richness of gut microbiomes increased with age from birth to about the third year, and then was much less variable, and (2) taxonomic composition of adult gut microbiomes from the Unites States differed strongly from those of Malawi and Venezuela, while the latter two showed less pronounced differences. In our analysis we do neither use richness (we normalize to a common richness), nor taxonomic labels, but we use solely the abundance vectors (Eq 1) as quantitative descriptors of NRAD shapes. Nevertheless, we will in the following show results consistent with key results from [31] with NRADs. Additionally, we will present a novel NRAD-based quantitative model for the development of gut microbiome entropy as function of age.

Prior to normalization, the richness of the samples covered three orders of magnitude, from 4105 to 296214 different ranks. All 528 RADs were normalized to the same MaxRank of *R* = 4105 to make RAD shapes and quantities computed thereof comparable.

#### NRADs differentiate between ages and countries

**Fig 7:**
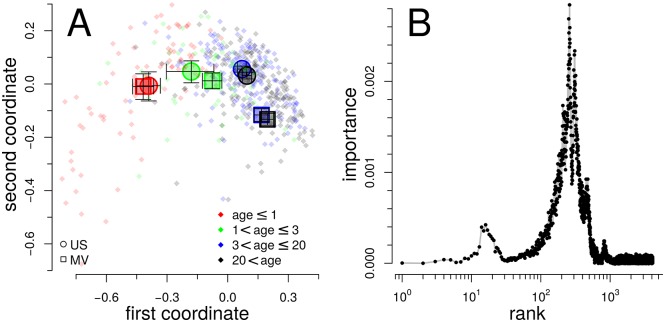
Country of origin and age as determinants of gut microbiomes NRADs. (A) MDS-ordination of NRADs of those 489 gut microbiomes from Malawi/Venezuela (MV) and United States (US) with age information. Small symbols represent individual NRADs, large symbols are averages. Error bars are 90% confidence intervals of the averages. The two coordinates of the MDS plot explain 83% of the NRAD distances. (B) Importance of each of the 4105 NRAD ranks for the random forest classification according to country of origin (MV vs. US). The two peaks around ranks 20 and 200 are the NRAD regions that carry most information about the country of origin.

First we applied MDS to the distance matrix of all 528 NRADs to see whether NRADs of different countries (especially between US = United States and MV = Malawi/Venezuela) and ages can be distinguished (Fig 7A). We found a banana shaped distribution in the MDS plot, arranged along the first coordinate that explains two thirds of the spread between NRADs (the banana shape also appears with nonmetric MDS [52]). The banana reaches from babies and small children on the left to adults on the right. Thus we have a clear age related trend of NRADs. It is remarkable that while the points for the smallest children have the largest scatter, the averages between MV and US are the same within the margin of error. Above the youngest age group, NRADs split up into two branches, one for MV and one for US. For adults the means of the MDS clusters of MV and US have small errors and are clearly separated. Differences between the older age groups within the same country are small. The described patterns in the MDS plot is consistent with the taxonomy based results in [31].

Yatsunenko *et al.* [31] had trained a statistical model that identified bacterial species characteristic to each of the countries. These species could be used to predict the country of origin from the taxonomic composition.

The pattern in the MDS plot (Fig 7A) suggests that it could be possible to train for the older age groups a statistical model that correctly predicts the country of origin of a sample from the shape of the NRAD. To test this hypothesis we trained a random forest model to classify NRADs from individuals older than 3 years according to country Malawi/Venezuela (MV, 89 NRADs) or United States (US, 254 NRADs). We found a high accuracy *ACC* = 0.94 ± 0.02 (mean ± standard error) of the model in threefold cross-validation. However, since the dataset is by far not evenly distributed over both countries, *ACC* could grossly overrate the performance. We therefore computed the *κ*-statistic and found *κ* = 0.85 ± 0.05, confirming the very good performance of this statistical model for predicting country of origin from NRAD. To avoid misunderstandings, we emphasize that “country of origin” is here a proxy for conditions, e.g. life style or diet, prevailing in a sample set that lead to a certain type of NRAD. If these conditions are similar, it is conceivable that NRADs will be similar too and therefore cannot be separated accurately.

It is interesting that not the head or tail region is most important for the classification performance, but that two separate regions in the middle carry most of the information about the country of origin (Fig 7B). Thus we could have normalized to even smaller *R* values without too much loss of information for the classification.

#### A quantitative model for the change of gut microbiome NRAD entropies with age

**Fig 8:**
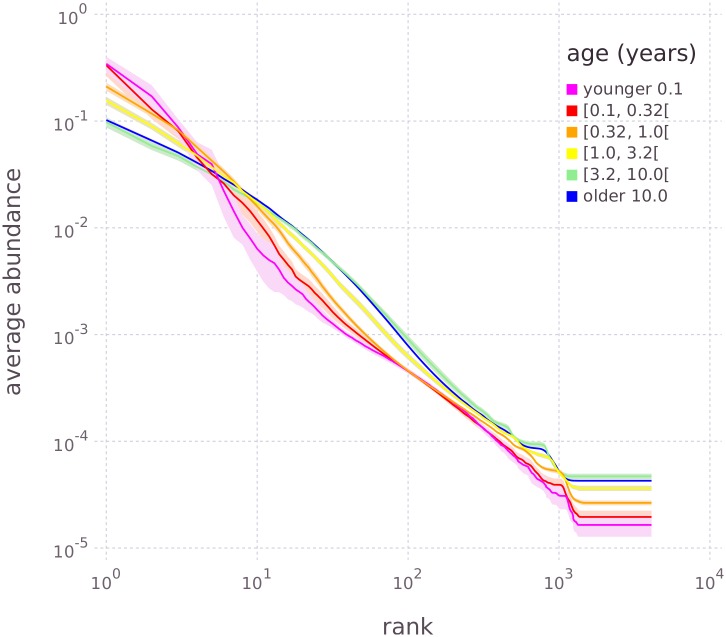
Averaged NRADs of gut microbiome data in six age groups. The number of NRADs per group from youngest to oldest were 9, 18, 55, 64, 34, and 309, respectively. Solid lines are mean NRADs, shaded areas are 90% confidence intervals for the means.

Yatsunenko *et al.* [31] observed that the taxonomic richness in gut microbiomes increased strongly during the first 3 years of age, and then stabilized. We asked whether NRADs reflect this dynamics, even though NRADs ignore taxonomy and eliminate richness. For the first analysis (Fig 8) we split the dataset into log-age intervals of approximately equal lengths, so that we have shorter age intervals at young age when most of the changes are expected, and longer age intervals in the more stable later regime. We then averaged all NRADs in these intervals, irrespective of country of origin.

The NRADs in Fig 8 have a clear dynamics with age: The average abundance in the head region up to about rank 10^0.6^ ≈ 4 decreases continuously from the youngest to the oldest age groups, while in the middle and tail regions the average abundance increases from youngest to oldest. These changes are fast in the youngest age groups, and slow down in the oldest groups that have practically the same NRADs. The change from NRADs with strong heads and weak tails in the young, to NRADs with weaker heads and stronger tails in the old means that the evenness of the distribution increases. Since evenness is one aspect of diversity [3], we conclude that the growing species richness described by [31] in their taxonomy-driven analysis is mirrored by an increase of evenness with age as computed from NRADs.

We now proceed from the qualitative comparison of NRADs in Fig 8 to a quantitative model of Shannon entropy as a function of age. Quantitatively, Shannon evenness *J*^(*i*)^ of NRAD *i* can be expressed in terms of entropy *H*^(*i*)^ and species richness *S*^(*i*)^ as [3]:

$$J^{\left(i\right)}=\frac{1}{\log S^{\left(i\right)}}\cdot H^{\left(i\right)}\;\;\;\text{with}\;\;\;H^{\left(i\right)}=-\sum_{r=1}^{R}a_{ir}\log a_{ir},$$ (5)

where we have used rank abundances *a*_*ir*_ according to Eq (1), and, for simplicity, plain symbols *J*, *H* instead of the commonly used *J*′, *H*′. Since by definition for MaxRank normalized RADs we have *S* = *R* = *const*, the evenness *J* computed from NRADs is proportional to the Shannon entropy *H* of these NRADs. Thus, we can rephrase our observation that NRAD evenness *J* grows with age as an increase of NRAD entropy *H* with age, or, because entropy is a measure of diversity, as growth of diversity with age.

Entropy is sensitive to sampling errors, and therefore not an ideal measure of diversity [3]. For instance, bigger samples from the same biological system have often higher richness which directly affects entropy. MaxRank normalization eliminates richness and thus attenuates this error. This property invites quantitative comparison of evenness or entropy across many samples. Here we exploit this to study the 489 HTSeq datasets of gut microbiomes that had information about age in their metadata. The richness in these samples varies over three orders of magnitude, between 4 × 10^3^ and 3 × 10^5^.

**Fig 9:**
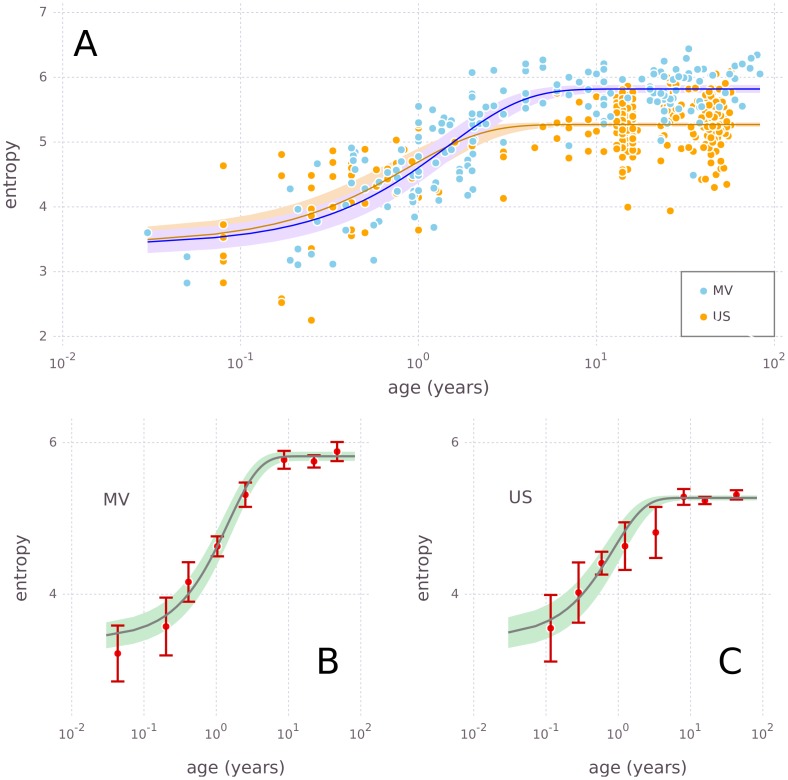
Development of gut microbiome entropy *H*_*R*_ with age *t*. (A) Entropies $H_{R}^{\left(i\right)}$ (with *R* = 4105) in nats for 181 samples from Malawi and Venezuela (MV, blue dots), and 308 samples from the United States (US, orange dots). Log-scaled horizontal axis is age in years. Superimposed are models for $H_{R}^{MV}\left(t\right)$ (blue line) and $H_{R}^{US}\left(t\right)$ (orange line) according to Eq (6). Areas around the model lines shaded in blue and orange are the corresponding 90% confidence intervals of the respective models. (B) and (C) Comparison of mean entropies of measured data (red points) and their corresponding 90% confidence intervals (error bars), with the model (solid gray lines) and its 90% confidence interval (shaded areas), for MV (panel B) and US (panel C). Model lines and shaded areas are the same as in panel A.

In Fig 9A entropies of the 489 gut microbiomes with age information are plotted against age. The dynamics of average entropy with age is consistent with Fig 8 discussed previously, namely a strong increase in the youngest and stabilization in older individuals.

The empirically observed change of entropy with age could be explained by a simple quantitative model. We assume that the youngest babies have a gut microbiome of very low diversity. Every microbial intake by the child will therefore have potentially a large impact on the diversity of its microbiome. This will lead to an increasing diversity of the gut microbiome with age. However, as the diversity increases, the impact of new intakes on the diversity will decrease since some of the species have already been taken up earlier. Thus we expect an increase that asymptotically approaches a diversity typical for the environment and life style of the individual. One of the simplest models for entropy *H*_*R*_ as measure of diversity with age *t* that shows this behavior is:

$$H_{R}\left(t\right)=H_{R}^{max}-\left(H_{R}^{max}-H_{R}^{0}\right)\;e^{-\lambda_{R}t},$$ (6)

with three parameters, the maximum entropy $H_{R}^{max}$ defining the asymptotic diversity, the entropy $H_{R}^{0}$ of the gut microbiome shortly after birth, and the entropy growth rate *λ*_*R*_. All quantities have an index *R* to remind us that we base our model on NRADs with a certain MaxRank *R*.

We have fitted two sets of optimal parameters, one with the MV and one with the US data (Table 1).

**Table 1:** Optimal parameters for the model of gut microbiome NRAD entropy as function of age.

	$H_{R}^{0}$	$H_{R}^{max}$	*λ*_*R*_ (*yr*^−1^)
*MV*	3.41 ± 0.17	5.82 ± 0.06	0.69 ± 0.09
*US*	3.43 ± 0.19	5.27 ± 0.03	1.16 ± 0.27

Parameters of optimal models Eq (6) fitted separately to the data from MV and US. Errors are 90% confidence intervals.

$H_{R}^{0}$ $H_{R}^{0}$ The corresponding models are the solid lines in Fig 9. Fig 9B and 9C show that the fitted models capture the average development of diversity with age and the differences between MV and US. The models have a reasonable accuracy with coefficients of determination of $r_{MV}^{2}=0.74$ and $r_{US}^{2}=0.52$. For all points in Fig 9B and 9C data and model are consistent, with one boundary case being the US model around age 3 *yr* (≈10^0.5^ *yr*), where it has a larger error because only a small number of measurements were available (see Fig 9A at this age).

The models are consistent with the observations by [31] in their taxonomy-based analysis, such as the increase of gut microbiome diversity in babies and children up to about three years, the split between MV and US in small children, and the turn to the higher asymptotic level in MV and the lower asymptotic level in US. Quantitatively, the model predicts that the youngest babies on average have gut microbiomes with *H*_4105_ ≈ 3.42 in both MV and US. The averages cannot be distinguished between MV and US up to about 2 *yr* (= 10^0.3^ *yr*). On average, children in the US reach the plateau $H_{4105}^{max,US}=5.27$ at about 3.2 *yr* (= 10^0.5^ *yr*), children in MV reach the higher plateau $H_{4105}^{max,MV}=5.82$ at about 5.6 *yr* (= 10^0.75^ *yr*). It must be emphasized that the model predicts *average* diversities as a function of age, not individual diversities. The large spread of entropy values around the average model in Fig 9A shows that inter-individual variation cannot be neglected. Finally, although the model is simple, plausible and fits the data well, other mathematical forms than Eq (6) are possible.

### GlobalPatterns dataset: Strengths and limitations of NRADs

Analyses with NRADs are complementary to taxonomy-based analyses: NRADs are blind to taxonomy, which is both a limitation and an advantage. It is a limitation because community biology is a function of taxonomic composition. It is an advantage because it enables quantitative comparison between taxonomically different generalized communities and thus discovery of generic community biology that is independent of actual taxonomic composition. A dataset where these aspects can be explored is the GlobalPattern dataset of Caporaso *et al.* [32] with 26 samples from human microbiomes, various environments, and mock communities. We transformed OTU tables to RADs and normalized them to MaxRank *R* = 2067, the minimum richness in the set, and we computed a distance matrix of all pairs of NRADs.

**Fig 10:**
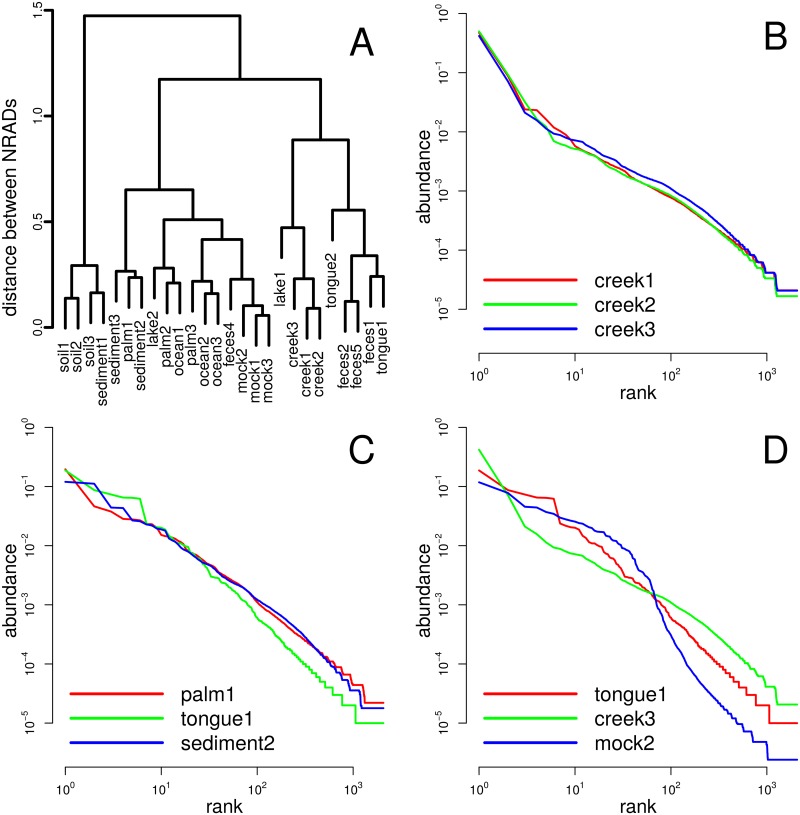
GlobalPatterns dataset. (A) Hierarchical clustering dendrogram based on distances between NRADs. (B) NRADs of three samples of similar origin that form a cluster. (C) Microbiome of human palm of human individual 1 clusters closely with sediments 2 and 3, but is more distant to tongue microbiome of individual 1. (D) Three differently shaped NRADs with same entropy.

Hierarchical clustering of the distance matrix (dendrogram in Fig 10A) led to close clustering of some samples that also form taxonomic clusters [32], namely the creek samples and a lake sample, the mock communities, or the human tongue and most feces communities. The NRADs in these clusters have low distances and thus are similar (e.g. Fig 10B).

Other relationships are more unexpected. The taxonomy-based analysis by [32] clusters tongue and palm microbiomes together and clearly separates human microbiomes from environmental samples. Conversely, NRADs of microbiomes of human palms do cluster closer with environmental samples than with microbiomes of tongues or feces. For instance, the NRAD *palm1* does not cluster with the microbiome *tongue1* of the same individual, but most closely with the sediment samples. NRADs of both *palm1* and *sediment2* have a lower abundance of rank 1 than tongue1 but more heavy tails (Fig 10C), were *palm1* and *sediment2* fit almost perfectly. Reasons for this unexpected clustering are not known. It could be that microbiomes in tongue and feces are more strictly controlled by the host body and the microbiome itself, while microbiomes on palms are more exposed to the environment.

In the GlobalPatterns dataset, entropies *H*_*R*_ range from 2.67 (*tongue2*) to 6.58 (*soil2*) with a median of 4.10 and a standard deviation of 1.06, and one may be tempted to explain the observed clustering by different entropies. However, such a simple approach is not successful here. For instance, the three NRADs in Fig 10D have almost the same entropies ($H_{R}^{creek3}=3.98$, $H_{R}^{mock2}=3.95$, $H_{R}^{tongue1}=3.91$), but have completely different shapes, and are members of different clusters. In general, the rich information content of an NRAD cannot be reduced to a single scalar quantity.

In the introduction we have mentioned that a theoretical possibility to quantitatively compare pairs of RADs of different richness (a strength of MaxRank normalization) is the use of the Kolmogorov-Smirnov statistic *D*, without RAD normalization. This corresponds to using the Chebyshev distance between the corresponding two cumulative distribution functions with different support. Although it is obvious that this approach treats the tail regions in a problematic way, it is unclear whether this problem is of practical relevance. To test this, we treated *D* as a distance and reran the hierarchical clustering with this distance. In fact, this *D*-based tree recovers some of the features of Fig 10A, but in general shows less biologically meaningful clusters (S1 Fig). We conclude that a *D*-based analysis is in practice no alternative to MaxRank normalization.

As mentioned in the introduction, one commonly used procedure for quantitative RAD comparisons is a parametric approach. Typically, a standard distribution such as the log-normal is fitted to a set of RADs and the comparison is then reduced to a comparison of the fitted parameters. We have tested the viability of this approach by fitting to the RADs of all GlobalPatterns samples five standard distributions, broken stick as null model, preemption, log-normal, Zipf, and Mandelbrot [41]. For most samples, with the exception of some soil and sediment samples, we found clear *qualitative* deviations from all five fitted distributions (S2 Fig), or, in other words, only few fitted distributions reflected the actual RAD shapes. This means that this parametric approach is in general not an option for quantitative analysis of HTSeq data. In contrast, with our non-parametric approach we can quantitatively compare all information-rich NRADs in a consistent and detailed way.

### Robustness of analyses based on MaxRank normalization

The key feature of MaxRank normalization is that it enables quantitative RAD comparisons by mapping RADs with diverse richness values to NRADs of a common richness *R*. It is clear that the lower *R*, the less information can be carried by NRADs. This could make analyses based on NRADs sensitive to *R*. To test this, we have tested how the choice of *R* affects NRAD based classification, NRAD distances, and NRAD entropies, as used in the previous sections.

First, we address the question whether NRAD based classification is sensitive to *R* using the classification of gut microbiomes described earlier. We use the same random forest classification and threefold cross validation as before to classify NRADs of gut microbiomes of individuals older than 3 years into MV (Malawi/Venezuela) or US (United States). The results for *R* = 4105, 1000, and 250 are summarized in Table 2.

**Table 2:** Accuracy of NRAD-based classification.

*R*	*ACC*	*κ*
4105	0.948 ± 0.018	0.862 ± 0.049
1000	0.947 ± 0.022	0.860 ± 0.057
250	0.917 ± 0.025	0.784 ± 0.064

Accuracy *ACC* and *κ* statistic of NRAD-based classification for country of origin of gut microbiome as function of MaxRank *R*.

The table shows a weak reduction in accuracy and *κ* statistic with decreasing *R* and increasing errors, both explainable by a loss of information stored in the NRADs with lower *R*. However, the reduction of *R* from 4105 to 1000 affects the accuracy of the classifier barely, and even the classifier computed from NRADs with *R* = 250 has still a good accuracy. The weak dependency is consistent with the importance plot (Fig 7B) where the regions of high importance cover two extended rank intervals that can be mapped down to NRADs with *R* = 250 or lower, though with some losses.

**Fig 11:**
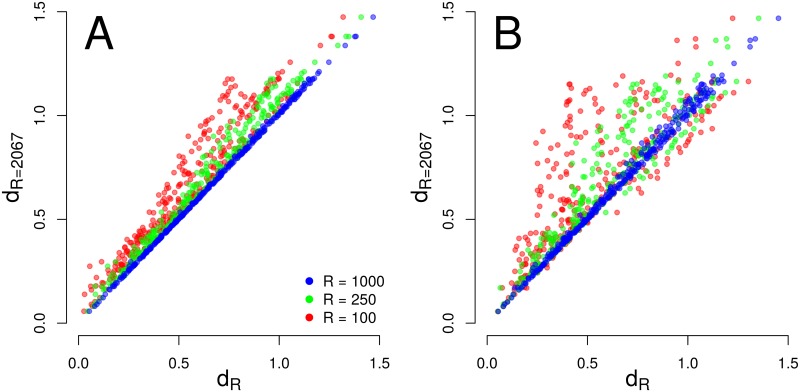
Dependence of NRAD distances *d*_*R*_ on MaxRank *R*. Co-ordinates are distances *d*_*R*_ between all 26 ⋅ (26 − 1)/2 = 325 NRAD pairs of the 26 GlobalPatterns samples at three different values of *R*. If the distance of an NRAD pair is the same for both *R*, the point lies on the diagonal. (A) MaxRank normalization; (B) cutoff normalization.

Secondly, we test the effect of *R* reduction on NRAD distances for the GlobalPatterns data set. Fig 11A demonstrates that NRAD distances (Eq 2) are well-conserved even if *R* is an order of magnitude lower than the richness of the original RADs. Approximately halving *R* from *R* = 2067 (the maximum possible *R* in the GlobalPatterns set) to *R* = 1000 does not affect distances between NRADs: the points are very close to the diagonal (coefficient of determination *r*^2^ = 1.000). If we reduce *R* more drastically to 250 (green points in Fig 11A), deviations appear, but we still have *r*^2^ = 0.976. The small deviations of NRAD distances in Fig 11A for *R* = 250 are all towards smaller NRAD distances (points shifted to the left of the diagonal), because lowering *R* from 2067 to 250 means that for *R* = 250 we straighten some of the fine-grained structure of NRADs with *R* = 2067 that allows for larger NRAD distances. Even for *R* = 100 we still have *r*^2^ = 0.913.

In Fig 11B we show for comparison how reduction of *R* affects NRAD distances for a simpler normalization scheme in which ranks above a given *R* are cut off (“cutoff normalization”), i.e. information in the tails with ranks higher than *R* is completely neglected. The scatter of the points is generally wider for all three values of *R* with *r*^2^ = 0.995, 0.874 and 0.614, respectively. These lower *r*^2^ values point to the importance of the tails that are neglected by cutoff normalization. NRAD pairs with differences predominantly in the neglected tails appear in Fig 11B shifted to the left of the diagonal, while NRAD pairs with more divergent heads and more similar tails appear right shifted. Cutoff normalization clearly is less robust than MaxRank normalization with respect to the choice of *R*.

As a consequence of the robustness of NRAD distances, the down-stream analyses that make use of NRAD distances are also quite robust. For instance, the hierarchical clustering (Fig 10A) of the GlobalPatterns dataset is identical between *R* = 250, *R* = 1000, and *R* = 2067 up to an agglomeration height of 0.98 (vertical axis in Fig 10A). Cluster assignments differ only above this agglomeration height at the highest branching points and thus at the most fuzzy cluster level.

Thirdly, we find for each sample *i* a regular and systematic *R* dependence of NRAD entropy $H_{R}^{\left(i\right)}$ (Eq 5):

$$H_{R}^{\left(i\right)}/\log R\approx c^{\left(i\right)}.$$ (7)

with a sample dependent constant *c*^(*i*)^. This approximation works reasonably well if *R* is not varied by more than an order of magnitude. Eq (7) corresponds formally to the definition of Shannon evenness with *R* interpreted as richness (Eq 5), i.e. Shannon evenness changes weakly with *R*. Moreover, this equation means that entropy or information content will systematically decrease with decreasing *R*. This decrease will depend weakly on *R* since log *R* changes only slowly with *R*.

As the NRAD entropies change systematically with *R*, this must also affect our model of entropy of gut microbiome NRADs as function of age. For instance if we approximately halve *R* from 4105 to 2000 and repeat the fitting of the model Eq (6) we arrive at $H_{2000}^{0,MV}=3.34\pm 0.16$, $H_{2000}^{0,US}=3.35\pm 0.19$, $\lambda_{2000}^{MV}=0.70\pm 0.09\;yr^{-1}$, $\lambda_{2000}^{US}=1.18\pm 0.27\;yr^{-1}$, $H_{2000}^{max,MV}=5.62\pm 0.06$, and $H_{2000}^{max,US}=5.12\pm 0.03$. Entropic model parameters *H*_2000_ are systematically lower by a small amount than the corresponding *H*_4105_ values (Table 1) as expected from an approximate scaling (Eq 7). If all entropic factors in Eq (6) scale in the same way, the scaling factor cancels, and the exponential growth term *e*^−*λt*^ should stay the same. In fact, the growth parameters *λ* are almost unaffected by the decrease of *R* from 4105 to 2000.

**Fig 12:**
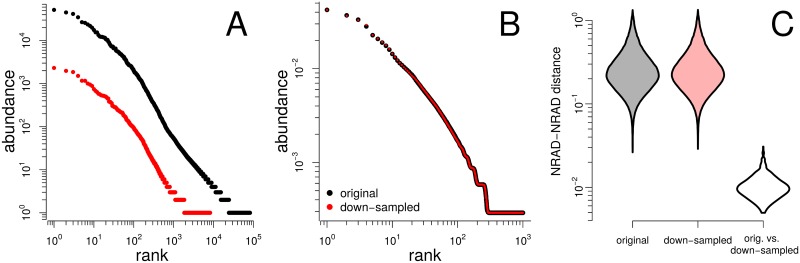
Robustness of NRADs against varying sampling depth. (A) original RAD of first sample of [31] (black) and down-sampled RAD (red). (B) the two NRADs obtained by MaxRank normalization to *R* = 1000 of the RADs in panel A are almost indistinguishable. (C) comparison of NRAD distances of the first 50 samples of the data set of [31]. Left violin plot: density of distances between NRADs computed by MaxRank normalization to *R* = 1000 of the original RADs; middle violin plot: same for down-sampled RADs; right violin plot: distances between corresponding original and down-sampled NRADs. The biologically meaningful NRAD distance distributions are robust against differences in sample size (left and middle violin). In comparison, the distances related to differences in sample size are negligible (right violin).

Finally, one important aspect of robustness is the following. If we take several samples of different size and therefore different richness of the same, well-mixed generalized community, then determine the RADs of those samples, and finally normalize these RADs to the same *R*, we should obtain the same NRAD for all samples. Only if this requirement is met can NRADs inform reliably about a generalized community. To test fulfillment of this requirement, we first down-sampled original HTSeq data sets by an order of magnitude in richness. Fig 12A shows as an example the original RAD and the down-sampled RAD of the first sample in the gut microbiome data of [31] (MG-RAST ID 4.4899263e6). We then normalized both to the same MaxRank *R* = 1000 (Fig 12B). For all samples we found that both RADs, the original and the down-sampled, led to practically the same NRAD. The violin plot Fig 12C makes a quantitative statement about this property: the biologically relevant distance distributions between NRADs are the same for both the original and down-sampled data (left and middle violin in Fig 12C are equal). In comparison, the distances between corresponding pairs of NRADs of original and down-sampled RADs are negligible (right violin in Fig 12C) as it should be for a normalization procedure that is robust against the size of the source sample.

In summary, we found that NRADs are robust quantitative descriptors of RADs. However, it is also clear that there are critical values of *R* below which essential RAD structures are lost and analyses become inconclusive. These critical values will depend on the studied RADs and on the analysis method. We recommend to monitor NRAD quality with methods suitable for the respective question. For instance, if the cluster structure of a set of NRADs is of interest, clustering and ordination methods as those presented above can be used to detect loss of structure when results for several *R* values are compared.

## Discussion

MaxRank normalization makes communities of different richness quantitatively comparable by mapping their RADs to NRADs of a common richness *R*. This is similar to projecting objects of different higher dimensions to one common lower dimension where the projections can be compared directly. The price to be paid is information loss, especially loss of richness information. However, the remaining information enables new approaches to community analysis. In particular, we could show that the information extracted from NRADs is sufficient to generate quantitative models of the dynamics or composition of generalized communities.

MaxRank normalization is not the only possible algorithm that maps higher richness RADs to a common lower richness, but it has crucial advantages over other procedures. For instance, another procedure is to cut off in all RADs ranks beyond a common maximum rank *R*. Obviously, this simple procedure neglects information in the RAD tails, leading to lower robustness as shown earlier. An alternative would be to scale the rank axis to the same maximum number. This would lead to fractional pseudo-ranks; the corresponding pseudo-rank abundance vectors have different dimensions and thus cannot be compared directly. Another alternative is the coarse-graining of the rank axis to a given number of pseudo-ranks. This is also not satisfying as the coarse-graining would treat samples of different richness differently, and because the pseudo-ranks are not observables. Conversely, MaxRank normalization is conceptually attractive since it corresponds to a real experimental sampling process with the attainment of a given richness as stop criterion. In fact, a similar observational protocol (“m-species list”), i.e. sampling up to a constant maximum rank, has been used in field ecology [53]. MaxRank normalization does not impose a specific model, i.e. the approach is model-free and generally applicable. For instance, we have applied it not only to HTSeq data but also to data from conventional ecological sampling of macro-invertebrates from fresh-water systems (unpublished).

MaxRank normalization (re-sampling up to a given maximum rank) may be confused with *rarefying* (re-sampling up to a given maximum number of individuals) [20, 22]. However, the two methods answer different diversity questions: rarefying allows answering questions that relate to sample richness (and typically also to OTU abundance), while NRAD comparison largely eliminates richness (and OTUs) and puts emphasis on abundance structure difference.

There are a number of limitations of MaxRank normalization. First, it is obvious that most information about richness is lost. Interestingly, it is not completely lost, but partially encoded in the NRADs, as a detailed technical analysis shows (to be published elsewhere). Nevertheless, if mainly changes or differences in richness are of interest, NRADs are not suitable.

Second, and related to the previous limitation, it is possible that some of the samples to be compared are richness-limited while others are not. In this case the information loss mentioned above can turn the method useless. To illustrate this point, imagine two systems that should be compared, one with two species, the other with thousands of species. In this case we would normalize the system to *R* = 2 and lose almost all the information in the RAD of the richer system. This limitation is usually not serious for the analysis of HTSeq data of generalized communities such as microbiomes. One strategy to cope with outlier samples of extremely low richness that would enforce the use of a low *R* and severe loss of information in NRADs is to discard such outliers, thus sacrificing some breadth for higher accuracy.

Third, a more severe problem with HTSeq data is the often implied assumption that read counts are quantitative measures of abundances. Unfortunately, HTSeq data can be biased by the experimental protocol, e.g. by preferential PCR amplification of certain species and non-amplification of others, or it can contain false positives, e.g. error mutants or chimeras produced in the experimental process [54, 55]. Since RADs are derived from OTU tables, any abundance bias that affects OTU tables will also affect RADs. Although these problems do not limit applicability of MaxRank normalization, they do limit the possible biological interpretation of the results. Related to this point, we found that changes in HTSeq protocols can significantly impact RADs and therefore NRADs. Thus, a stringent control is required if results from different studies are to be compared. Such a control can be implemented e.g. by comparing RADs resulting from the application of the different protocols to common reference samples. As HTSeq technology develops rapidly, some of these experimental problems may be solved in the near future. But even with these imperfections, NRADs can be used as generic quantitative descriptors to discover new community biology.

Fourth, analyses based on NRADs alone are blind to taxonomic composition. This can be an advantage because in this way generic effects that influence the abundance structure can become clearly visible. But this blindness to taxonomy makes NRAD based analyses inadequate if differences or changes in taxonomic composition are of major interest.

## Supporting Information

S1 Fig Kolmogorov-Smirnov statistic *D* as RAD-RAD distance. Hierarchical clustering dendrogram of GlobalPatterns RADs, using Kolmogorov-Smirnov statistic *D* between pairs of non-normalized RADs as distance (results with the Anderson-Darling statistic were essentially the same). Some of the samples (e.g. *soil1* and *soil2*) are clustered similar to the NRAD-NRAD distance based dendrogram Fig 10A of main text. More often, clustering based on NRAD distances is biologically more meaningful than *D*-based clustering (e.g. *ocean1/2/3*). In some cases, the *D*-based clustering is biologically outright wrong as for the cluster formed by the high-evenness mock samples and the low-evenness tongue samples.
(PDF)

S2 Fig Fits of four standard distributions to RADs of GlobalPatterns set. (PNG)

S1 Table Meta-data for GlobalPatterns and correspondence of original sample IDs and new sample names. (PDF)
