# Supplementary figures and images for: Quantitative Comparison of Abundance Structures of Generalized Communities: From B-Cell Receptor Repertoires to Microbiomes
Source: PLoS Comput Biol. 2017 Jan 23;13(1):e1005362. doi: 10.1371/journal.pcbi.1005362 (PMC5293285; doi:10.1371/journal.pcbi.1005362)

Kolmogorov-Smirnov statistic between RADs

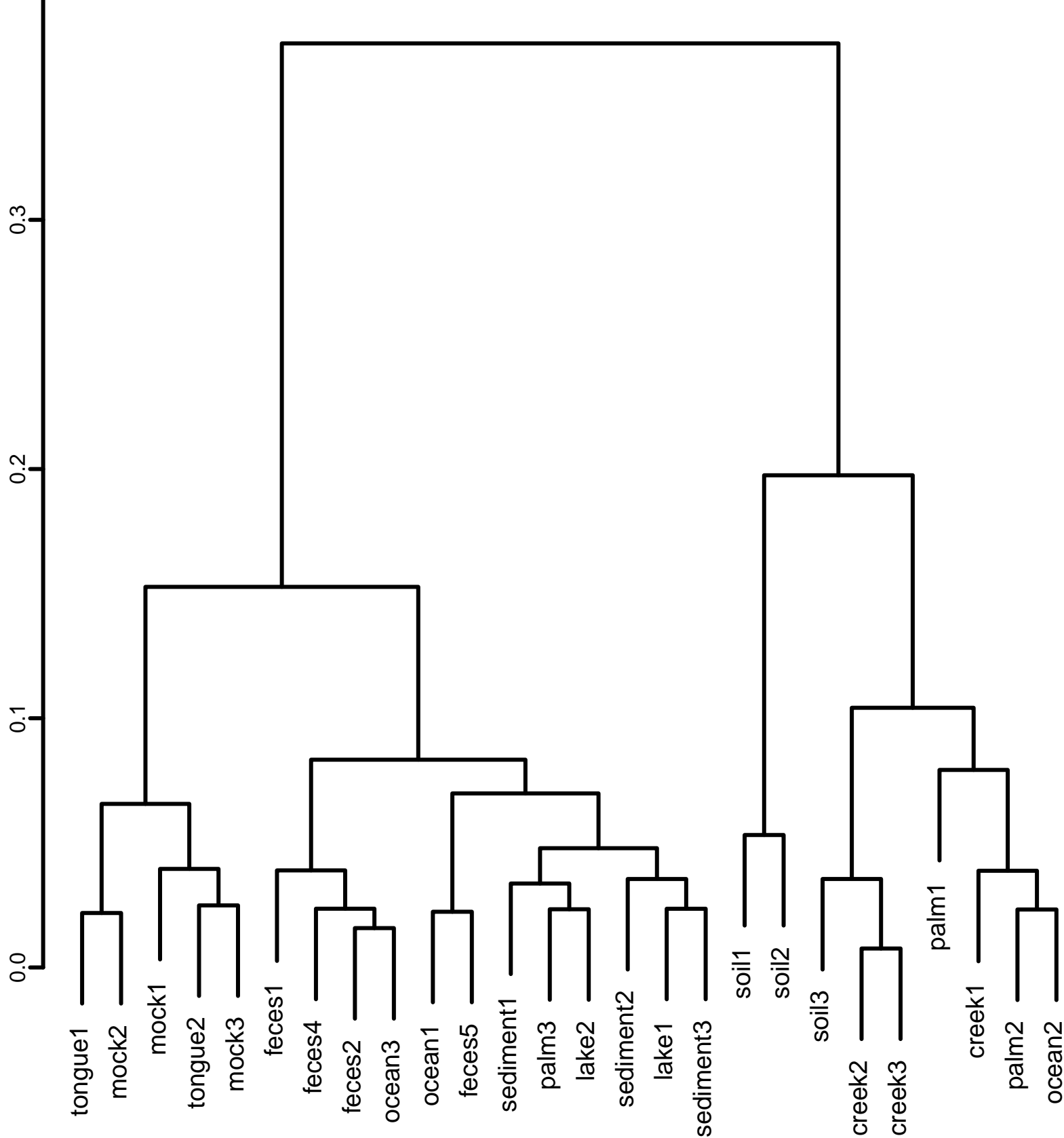

Supplement: S1 Fig — Hierarchical clustering dendrogram of GlobalPatterns RADs, using Kolmogorov-Smirnov statistic D between pairs of non-normalized RADs as distance (results with the Anderson-Darling statistic were essentially the same). Some of the samples (e.g. soil1 and soil2) are clustered similar to the NRAD-NRAD distance based dendrogram Fig 10A of main text. More often, clustering based on NRAD distances is biologically more meaningful than D-based clustering (e.g. ocean1/2/3). In some cases, the D-based clustering is biologically outright wrong as for the cluster formed by the high-evenness mock samples and the low-evenness tongue samples. (PDF) [file pcbi.1005362.s001.pdf]

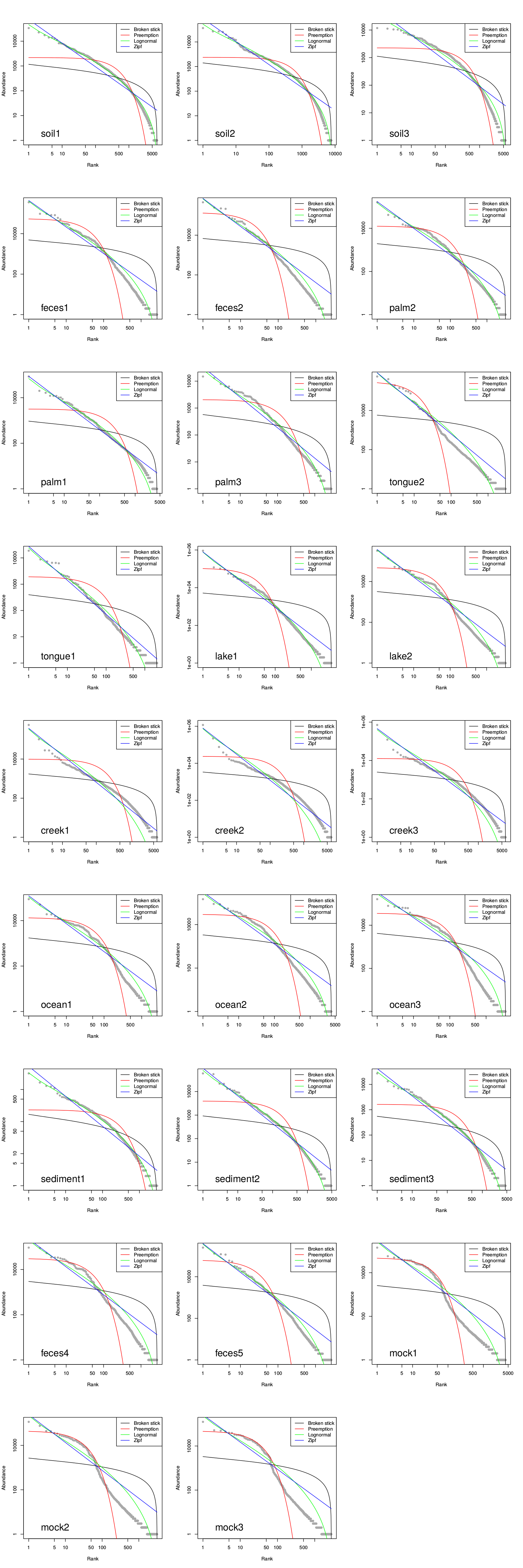

Supplement: S2 Fig — (PNG) [file pcbi.1005362.s002.png]
