# Supplementary material for: Quantitative Comparison of Abundance Structures of Generalized Communities: From B-Cell Receptor Repertoires to Microbiomes
Source: PLoS Comput Biol. 2017 Jan 23;13(1):e1005362. doi: 10.1371/journal.pcbi.1005362 (PMC5293285; doi:10.1371/journal.pcbi.1005362)
Supplement: S1 Table — (PDF) [file pcbi.1005362.s003.pdf]

	Sample ID	Sample Type	Description	Sample Name
1	CL3	Soil	Calhoun South Carolina Pine soil, pH 4.9	soil1
2	CC1	Soil	Cedar Creek Minnesota, grassland, pH 6.1	soil2
3	SV1	Soil	Sevilleta new Mexico, desert scrub, pH 8.3	soil3
4	M31Fcsw	Feces	M3, Day 1, fecal swab, whole body study	feces1
5	M11Fcsw	Feces	M1, Day 1, fecal swab, whole body study	feces2
6	M31Plmr	Skin	M3, Day 1, right palm, whole body study	palm2
7	M11Plmr	Skin	M1, Day 1, right palm, whole body study	palm1
8	F21Plmr	Skin	F1, Day 1, right palm, whole body study	palm3
9	M31Tong	Tongue	M3, Day 1, tongue, whole body study	tongue2
10	M11Tong	Tongue	M1, Day 1, tongue, whole body study	tongue1
11	LMEpi24M	Freshwater	Lake Mendota Minnesota, 24 meter epilimnion	lake1
12	SLEpi20M	Freshwater	Sparkling Lake Wisconsin, 20 meter eplimnion	lake2
13	AQC1cm	Freshwater (creek)	Allequash Creek, 0-1cm depth	creek1
14	AQC4cm	Freshwater (creek)	Allequash Creek, 3-4 cm depth	creek2
15	AQC7cm	Freshwater (creek)	Allequash Creek, 6-7 cm depth	creek3
16	NP2	Ocean	Newport Pier, CA surface water, Time 1	ocean1
17	NP3	Ocean	Newport Pier, CA surface water, Time 2	ocean2
18	NP5	Ocean	Newport Pier, CA surface water, Time 3	ocean3
19	TRRsed1	Sediment (estuary)	Tijuana River Reserve, depth 1	sediment1
20	TRRsed2	Sediment (estuary)	Tijuana River Reserve, depth 2	sediment2
21	TRRsed3	Sediment (estuary)	Tijuana River Reserve, depth 2	sediment3
22	TS28	Feces	Twin #1	feces4
23	TS29	Feces	Twin #2	feces5
24	Even1	Mock	Even1	mock1
25	Even2	Mock	Even2	mock2
26	Even3	Mock	Even3	mock3

Table 1: Meta-data for GlobalPatterns and correspondence of original sample IDs and new sample names.
